# A meta-analysis of the lymphatic microvessel density and survival in gastric cancer with 1809 cases

**DOI:** 10.18632/oncotarget.23526

**Published:** 2017-12-21

**Authors:** Liang Liang, Wen-Ting Huang, Rong-Quan He, Hai-Wei Liang, Chun-Qin Huang, Hong Zhou, Fang-Lin Wei, Sheng-Sheng Zhou, Zhi-Gang Peng, Gang Chen, Jun-Qiang Chen, Xin-Gan Qin

**Affiliations:** ^1^ Department of Gastrointestinal Surgery, First Affiliated Hospital of Guangxi Medical University, Nanning, Guangxi Zhuang Autonomous Region, People's Republic of China; ^2^ Department of Pathology, First Affiliated Hospital of Guangxi Medical University, Nanning, Guangxi Zhuang Autonomous Region, People's Republic of China; ^3^ Department of Medical Oncology, First Affiliated Hospital of Guangxi Medical University, Nanning, Guangxi Zhuang Autonomous Region, People's Republic of China

**Keywords:** lymphatic microvessel density, gastric cancer, prognosis, meta-analysis

## Abstract

Lymph node metastasis commonly occurs in gastric cancer. Previous studies have demonstrated that the overexpression of lymphatic microvessel density (LVD) is correlated with various malignancies. To evaluate the potential role of LVD in various malignancies, we conducted a systematic review and meta-analysis to thoroughly investigate the association of LVD expression with tumor progression and survival in gastric cancer. We performed a comprehensive search of common databases and selected studies demonstrating the relationship between LVD expression and gastric cancer prognosis. Hazard ratios (HR) were used to determine the value of LVD for predicting gastric cancer metastasis and prognosis. The data were extracted from the included studies and pooled with the appropriate effects model using STATA 12.0. The results showed that high LVD expression obviously impacted the prognosis of gastric cancer, based on an overall survival (OS) HR of 2.58 (95% CI: 1.91–3.48, *P* < 0.001) and a disease-free survival (DFS) HR of 2.51 (95% CI: 1.35–4.68, *P* = 0.004) in the univariate analysis. In addition, the results of the multivariate analysis indicated a remarkable relationship between high LVD expression and gastric neoplasm prognosis. The pooled OS HR was 4.12 (95% CI: 3.45–4.91, *P* < 0.001). The current meta-analysis shows that high LVD is closely related to tumor metastasis and poor prognosis in gastric malignancy. LVD could be a key factor in tumor lymphatic metastasis. Moreover, LVD is likely a potential index and an effective biomarker for the prediction of patient prognosis.

## INTRODUCTION

Cancer is currently the second most common cause of death in the United States, and it is expected to surpass heart disease as the leading cause of death in the coming years [1]. Despite a decreased incidence in recent decades, gastric cancer remains a primary public health concern worldwide [2]. Approximately half of all patients diagnosed with gastric cancer show advanced disease and a poor 5-year survival rate of less than 20% [3, 4]. Based on these statistics, the high death rate of cancer has attracted public attention to the diagnosis and treatment of this disease, and it is of paramount importance to identify factors that can efficiently predict survival and response to treatment to help select better therapeutic tools from among the available resources [3].

Tumor metastasis is a complex process in malignant neoplasms. It includes three phases: adhesion, degradation and migration. Vasculogenesis and lymphangiogenesis are important in the development, growth, invasion and metastasis of tumors. The molecules of tumor cells or stroma cells can stimulate lymphatic vessel formation, thereby facilitating tumor cell invasion. This process of lymphangiogenesis occurs prior to metastasis, creating a favorable microenvironment for disseminating primary tumor cells [5]. Additionally, when tumor cells penetrate into peri-tumoral lymphatic vessels, they may promote or enhance lymphangiogenesis by stimulating the proliferation of normal lymphatic endothelial cells [6]. It has been reported that microvessels provide nutrition for neoplasm growth, and further studies have shown that lymphatic vessel growth is crucial for metastasis [7]. The LVD represents the lymphangiogenesis rate. The discovery and application of antibody markers of lymphatic endothelial cells make lymphangiogenesis and tumor metastasis easier to assess.

The lymphatic vessel system plays an important role in lymph node metastasis, and malignant cells that spread to regional lymph nodes during the early stages of tumor dissemination rely on the lymphatic vasculature [8]. It has been reported that high-density lymphatic vessels and lymphangiogenesis promote the spread of neoplasm cells, leading to an unfavorable prognosis and decreased overall survival of patients with non-small cell lung cancer [9]. This theory is based on the fact that the LVD can be used to quantify tumor lymphangiogenesis through immunohistochemistry using D2-40 staining. Thus, we conducted this meta-analysis to quantify the association between LVD and prognosis in gastric cancer to provide a valuable index for clinical prognostic assessment.

Podoplanin is a 38-kd mucin-like transmembrane glycoprotein that is specifically and highly expressed in lymphatic endothelial cells. D2-40, as an available antibody, can specifically recognize human podoplanin with similarities to the M2A antigen [10, 11]. D2-40 has been reported to react with the oncofetal antigen, which is expressed in the fetal testis and on the surface of testicular germ cell tumors rather than in the adult testis [12]. D2-40 is widely used in clinical tissue specimens as a selective and effective marker to show microvessel, but not blood vessel, endothelium [13]. Lymphatic vessel endothelial receptor 1 (LYVE-1) is a receptor for the extracellular matrix/lymphatic fluid glycosaminoglycan, and it can also serve as a lymphatic endothelium marker for visualizing the neoplasm's lymphatic microvessels through immunohistochemistry [14].

## RESULTS

### Characteristics of the included studies and systematic reviews

**Figure 1:**
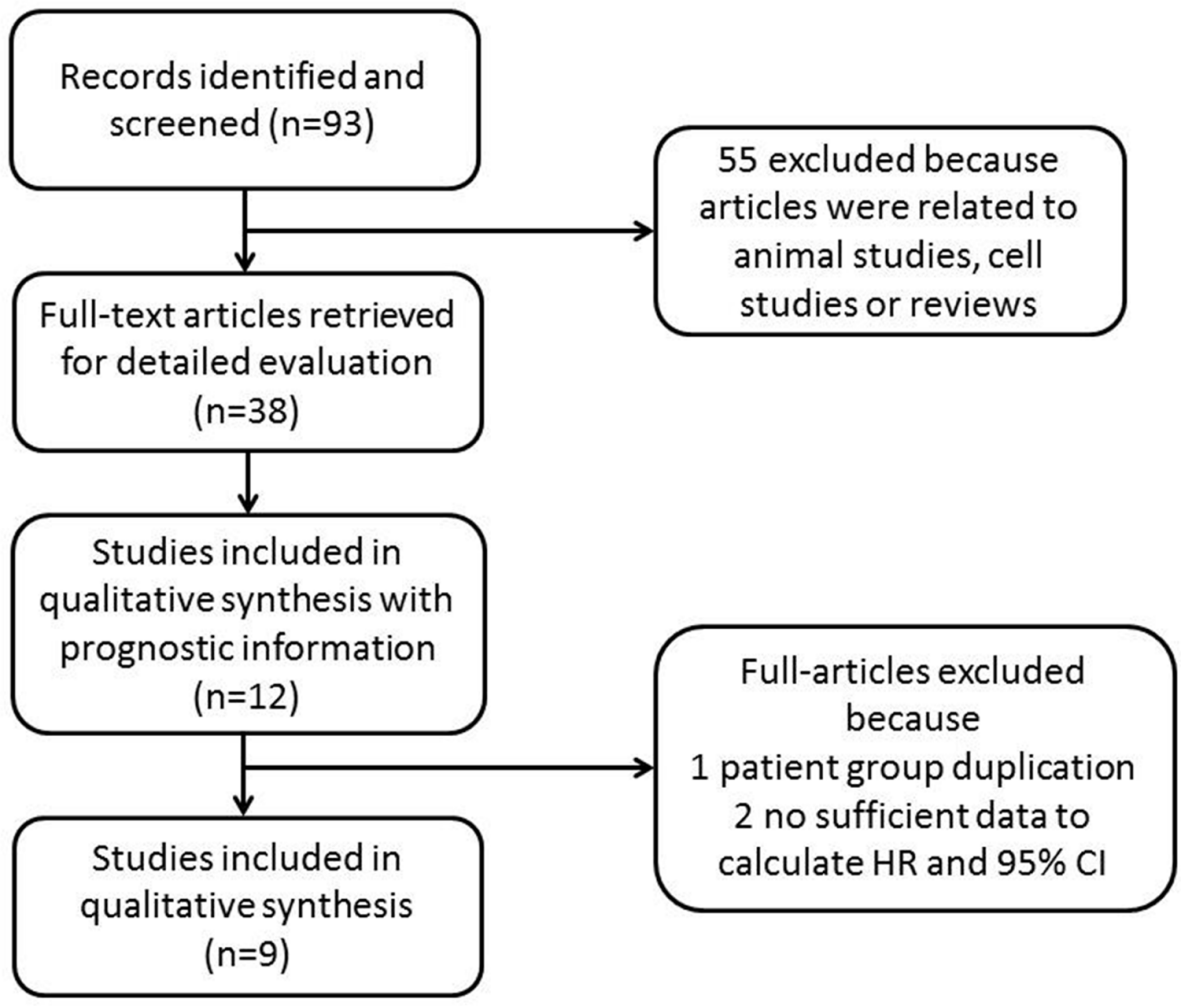
Flow chart of the literature search and study selection

During the initial search, ninety-three studies were retrieved concerning LVD in gastric cancer, including 44 studies from English databases and 49 studies from Chinese databases. After relevant titles and abstracts were screened, twelve studies met the inclusion criteria of reporting the prognostic characteristics of LVD for the survival of gastric cancer patients. Ten studies were written in English, and two studies were written in Chinese, with publication years ranging from 2006 to 2015. Two studies were ineligible for evaluation in this meta-analysis as they did not provide sufficient data to estimate HRs and variances, and although we tried to contact the authors, there was no response. One article duplicated an existing patient group. Thus, 9 studies were included in this meta-analysis (Figure 1). The characteristics of the 9 eligible full-text studies are listed in Table 1 [15–23]. A total of 1809 patients were included in the systemic review. The sample sizes of the studies varied from 56 to 1072, with a median of 72. As Table 1 shows, the IHC method using the D2-40 antibody was used to analyze LVD. Notably, Gao P *et al.* [17] and Liu *et al.* [23] used the LYVE-1 antibody. One to 5 hotspot areas were selected for higher magnification, and positive vessels were counted. High LVD reactivity was distinguished from low LVD reactivity based on cut-off values that varied among the studies. Newcastle-Ottawa Quality Assessment Scale (NOS) scores are shown in Table 2, and all studies met the established score standard.

**Table 1:** Main characteristics and results of the eligible studies

First author	Year	Country	Number	Antibody type	Hotspots selected	Magnification field	Cut-off value	HR statistics	Univariate analysis	HR (95% CI)	Multivariate analysis	HR (95% CI)	Reference
Nakamura Y	2006	Japan	117	D2-40	5	×200	12	SC, reported	OS	2.84 (1.2–6.74)	OS	2.49 (1.03–5.99)	[15]
Cao F	2013	China	1072	D2-40	1^a^	×200	median	reported	NR	NR	OS	4.21 (3.48–5.08)	[16]
Gao P	2008	China	168	LYVE-1	3	×400	median	SC, reported	OS	NR	OS	6.24 (1.55–25.10)	[17]
									RFS	NR	RFS	6.57 (1.57–27.53)	
Ikeda K	2014	Japan	72	D2-40	5	×200	22	SC	OS	1.655 (0.69–3.96)	NR	NR	[18]
Yu JW	2011	China	68	D2-40	5	×300	14	SC	OS	3.19 (1.10–9.19)	NR	NR	[19]
Coşkun U	2009	Turkey	65	D2-40	3	×200	5	SC	OS	2.3 (1.04–5.11)	NR	NR	[20]
									DFS	2.34 (1.04–5.28)	NR	NR	
Pak KH	2015	South Korea	66	D2-40	5	×200	mean	SC	OS	2.52 (0.87–7.26)	NR	NR	[21]
									DFS	2.78 (1.06–7.28)	NR	NR	
Gou HF	2011	China	56	D2-40	5	×400	9.24	reported	OS	3.6 (1.68–7.71)	OS	4.29 1.78–10.36) (RR)	[22]
Liu XL	2013	China	125	LYVE-1	3	×200	CS	SC, reported	OS	2.49 (1.44–4.30)	OS	3.42 (1.21–7.82) (RR)	[23]

Abbreviations: HR: hazard ratio; CS: complex score; SC: survival curves; OS: overall survival; DFS: disease free survival; RFS: relapse free survival; NR: not report; RR: risk ratio; ^a^: area with the highest density.

**Table 2:** Study quality assessment (Newcastle-Ottawa Scale)

Study	Selection (score)				Comparability (score)	Exposure (score)			Total (score)
	Representativens of the exposed cohort	Selection of the non-exposed cohort	Ascertainment of exposure	Outcome of interest was not present at start of study	Control for important factor^a^	Assessment of outcome	Follow-up long enough for outcomes to occur	Adequacy of follow-up of cohorts	
Nakamura Y	*	*	*	*	-	*	*	*	7
Cao F	*	*	-	*	-	*	*	*	6
Gao P	*	*	-	*	-	*	*	*	6
Ikeda K	*	*	*	*	-	*	*	*	7
Yu JW	*	*	*	*	-	*	*	-	6
Coşkun U	*	*	-	*	-	*	*	*	6
Pak KH	*	*	-	*	-	*	*	*	6
Gou HF	*	*	*	*	-	*	*	*	7
Liu X	*	*	-	*	-	*	*	*	6

a A maximum of 2 stars can be allotted in this category, one for age, the other for other controlled factors

### Univariate analysis of survival status

**Figure 2:**
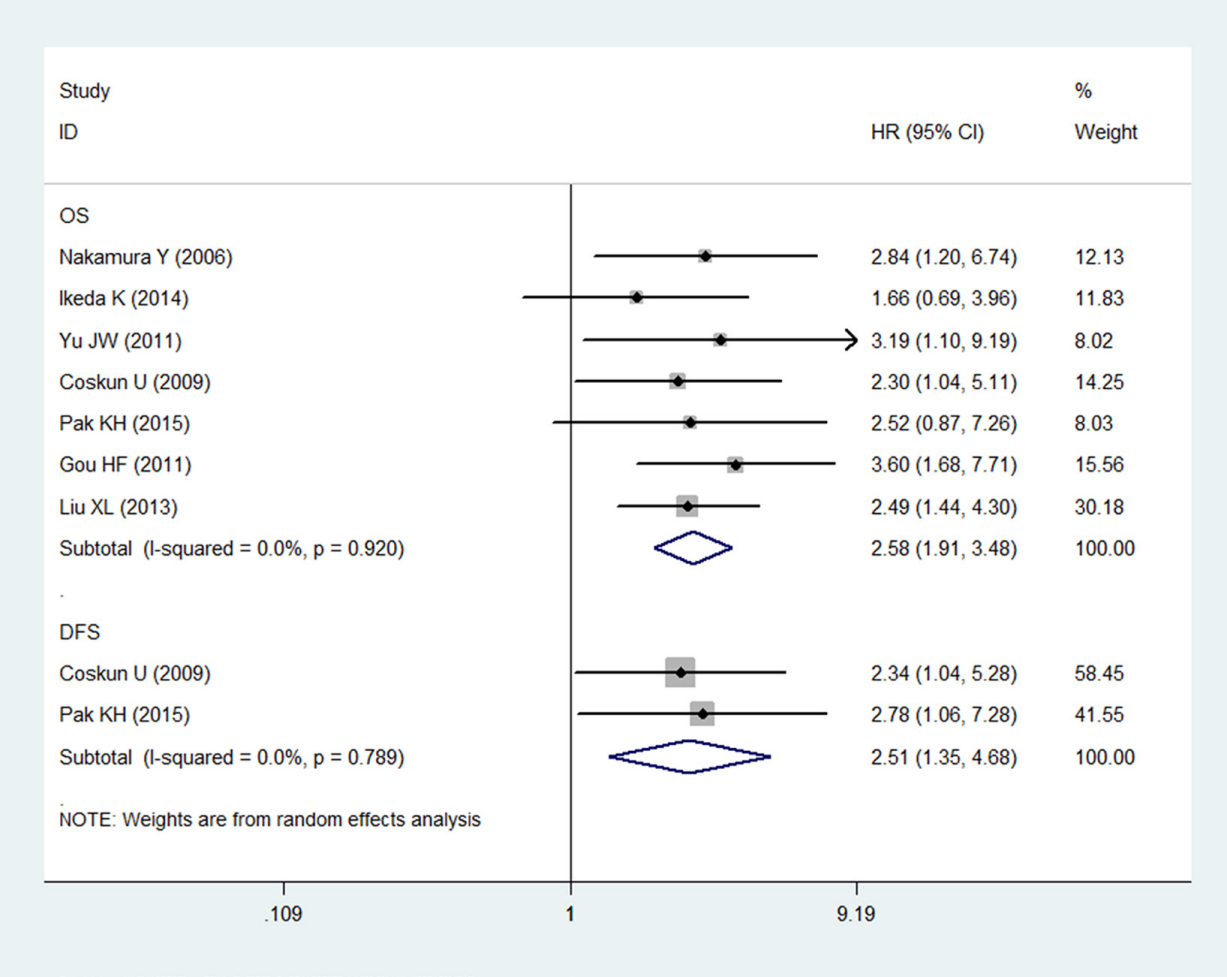
Forest plot showing the association between the LVD and OS/DFS in gastric cancer in the univariate analysis

In terms of the univariate analysis, 7 studies (*n* = 569) were available for our meta-analysis. Among these, 2 (*n* = 131) were included in the analysis of DFS, while all 7 studies (*n* = 569) were included in the OS analysis. No heterogeneity existed in the meta-analysis of DFS (*P* = 0.770, *I*^2^ = 0%) or OS (*P* = 0.919, *I*^2^ = 0%). Consequently, the random-effects model was applied to estimate the combined HRs. The combined HRs for OS and DFS were 2.58 (95% CI: 1.91–3.48, *P <* 0.001; Figure 2) and 2.51 (95% CI: 1.35–4.68, *P* = 0.004; Figure 2), respectively, suggesting that a high LVD was associated with worse DFS and OS in gastric cancer patients.

### Multivariate analysis of patient survival status

**Figure 3:**
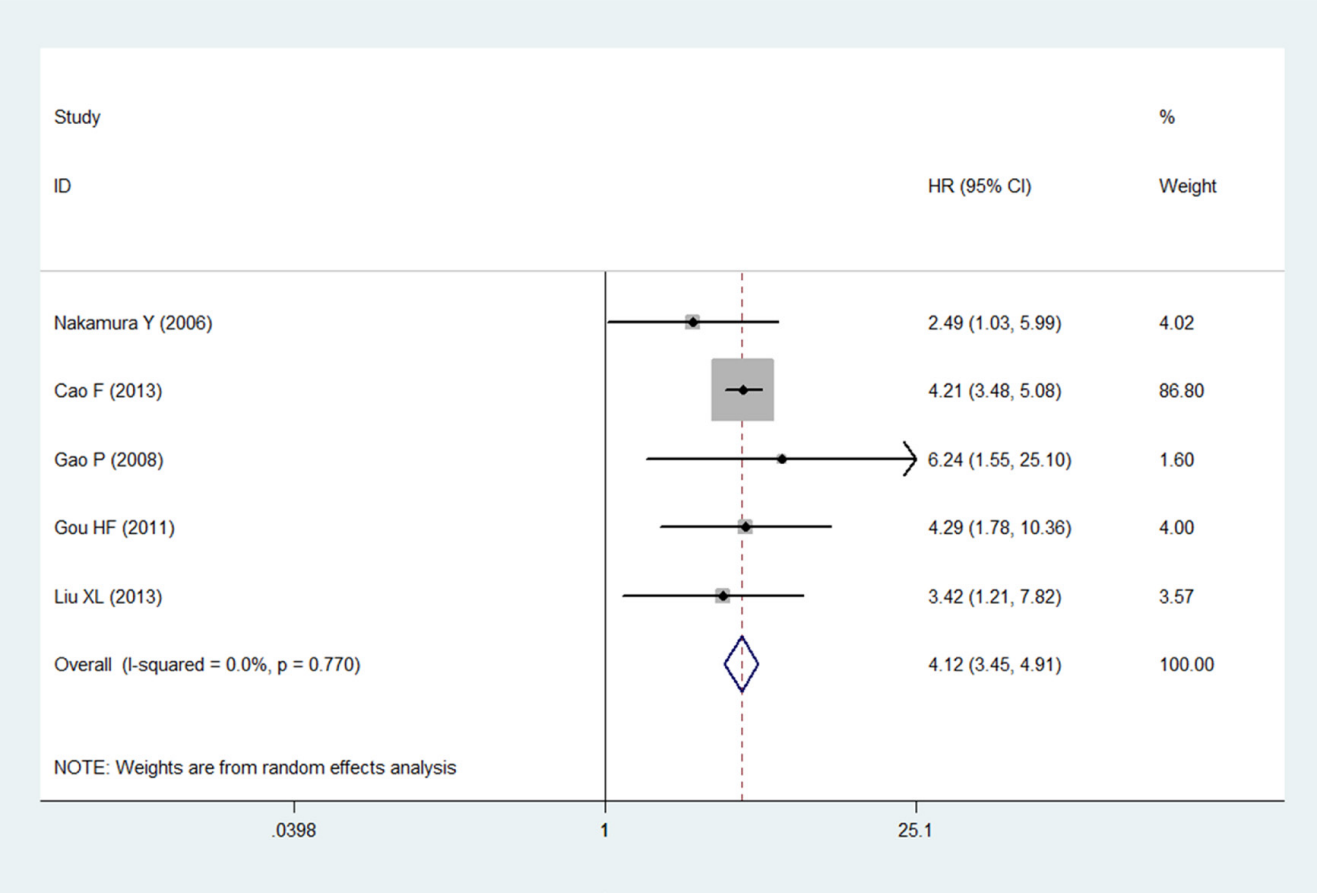
Forest plot showing the association between the LVD and OS in gastric cancer in the multivariate analysis

The multivariate analysis included six studies that measured the relationship between LVD and prognosis. Five of them found high LVD in relation to OS in gastric cancer. Only Guo P *et al.* [17] discussed the relationship between RFS and LVD. None of these studies analyzed DFS. Five studies with 1538 patients were eligible for inclusion in the meta-analysis to examine the combined HR for OS. The random-effects model was consistent with no heterogeneity for OS (*P* = 0.770, *I*^2^ = 0%). Thus, high LVD was indicative of an unsatisfactory clinical outcome, with a pooled HR of 4.12 (95% CI: 3.45–4.91, *P <* 0.001; Figure 3).

### Sensitivity analysis and publication bias

**Figure 4:**
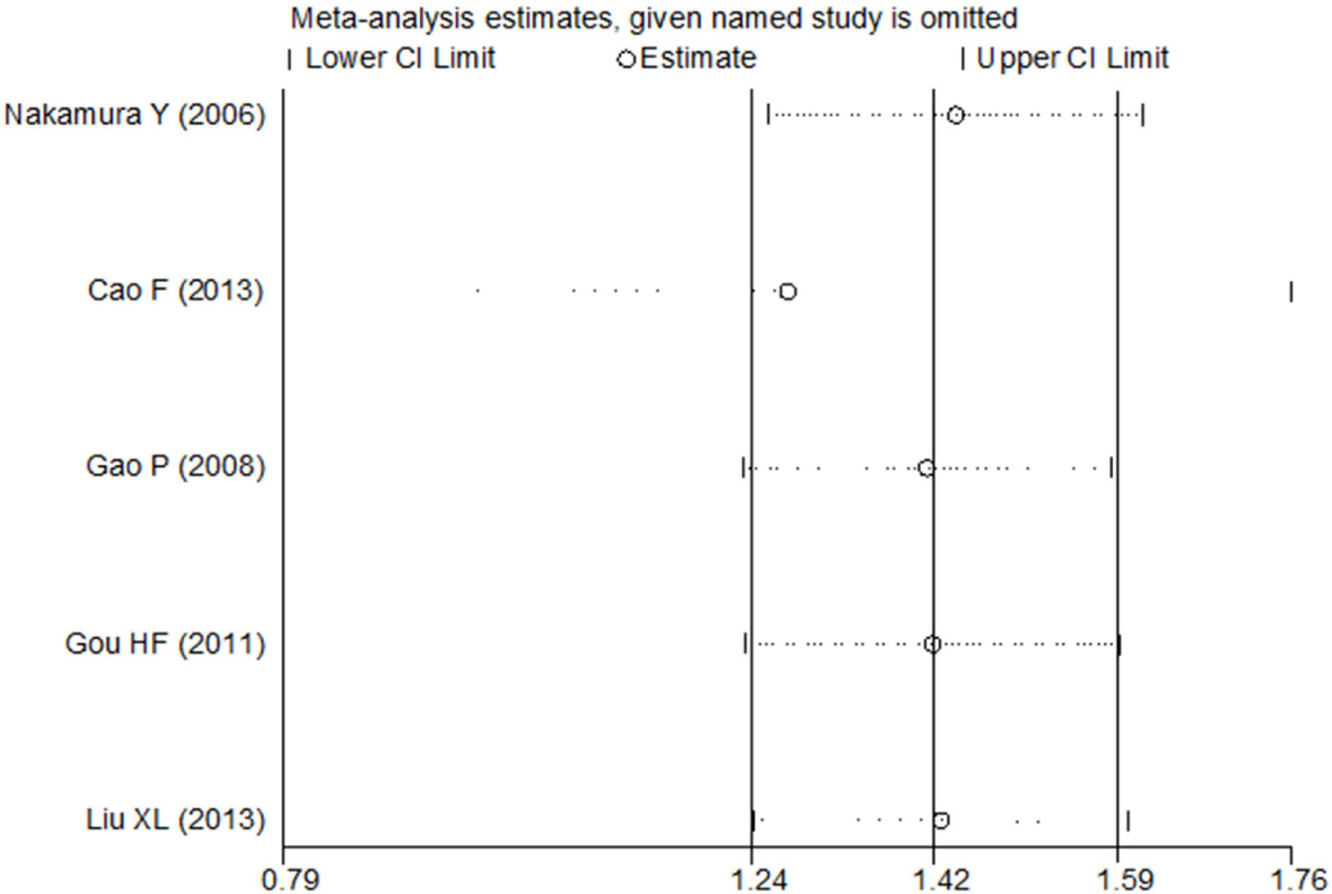
Sensitivity analysis of the multivariate analysis of five studies for OS

**Figure 5:**
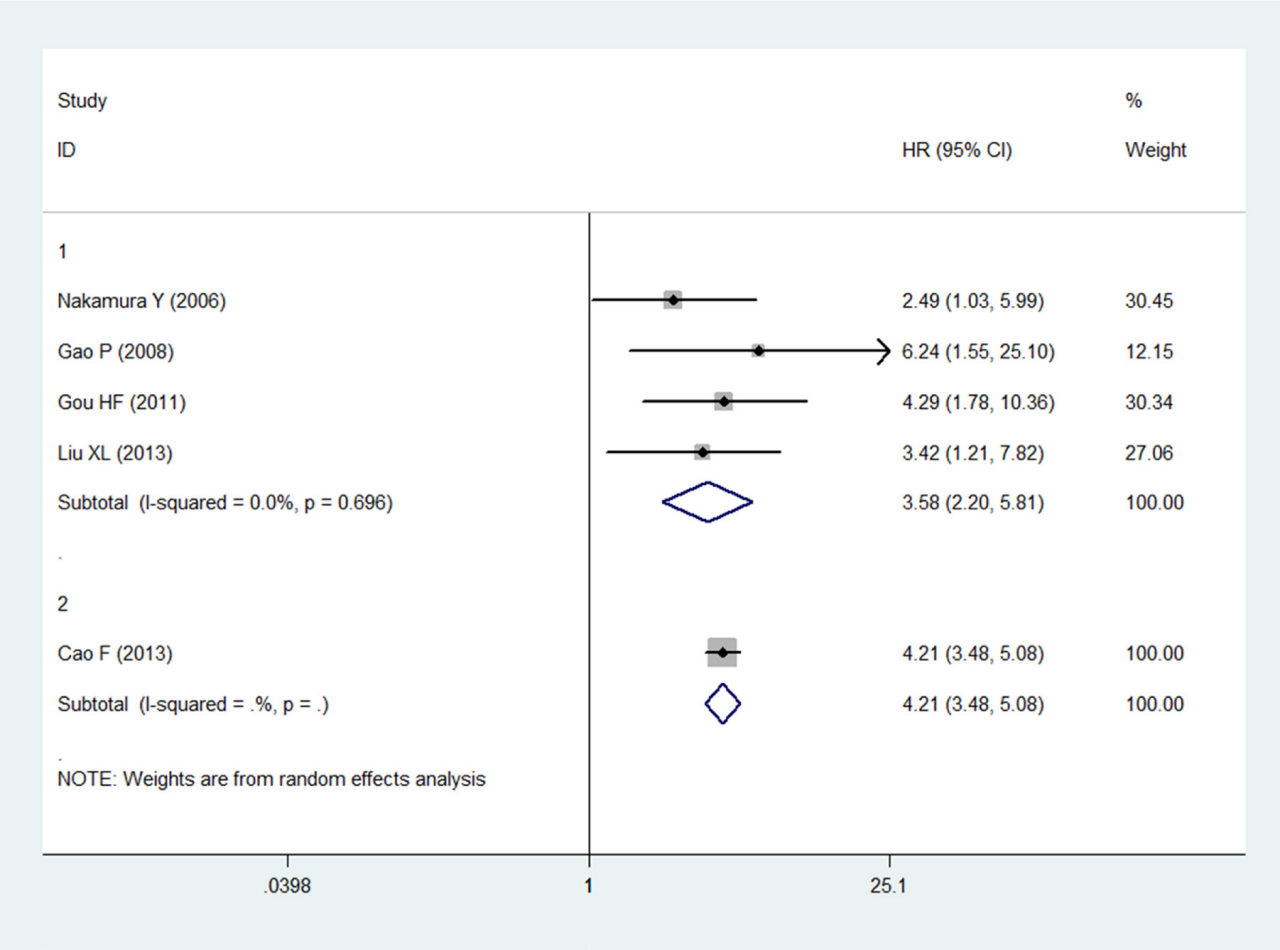
Forest plot showing the association between LVD and OS in the subgroup analysis

**Figure 6:**
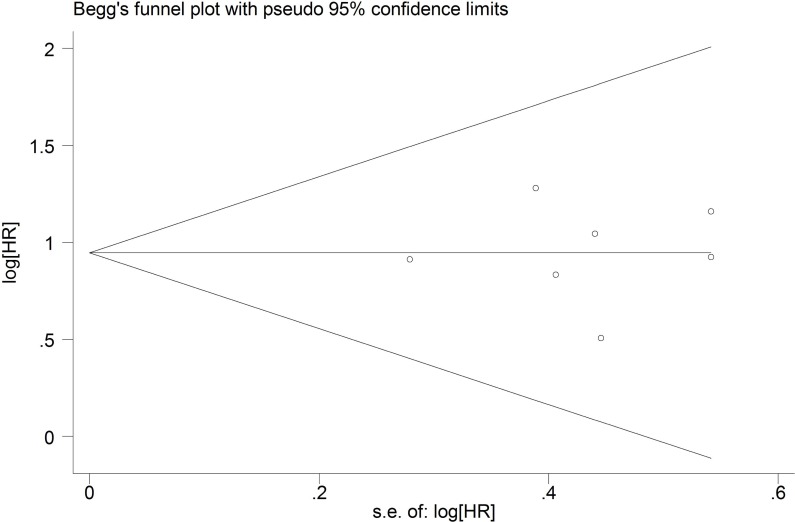
Begg’s funnel plot for publication bias based on the results of the univariate analysis

As no heterogeneity was observed in the present study, we did not perform a sensitivity analysis to assess the stability of the meta-analysis in the univariate analysis. However, the study by Cao F *et al.* [16] included a considerable number of patients, resulting in a hazard ratio weight of 86.8% for OS in the multivariate analysis. The results of the sensitivity and subgroup analysis for OS in the multivariate analysis are shown in Figures 4 and 5. The pooled estimate of the HRs (3.58, 2.20–5.81, *P* < 0.001) was significant within the scope of a 95% CI when the Cao F *et al.* study was removed. Publication bias statistics were determined using Begg's test with a funnel plot. For the univariate analysis, no significant asymmetrical distributions were observed in the OS group (*P* > 0.05; Figure 6). We did not detect a publication bias in the DFS group, possibly because of the small number of studies included. In the multivariate analysis, the results of the Begg's test using a funnel plot showed no evidence of a publication bias (*P* > 0.05).

## DISCUSSION

The current study examined nine studies with 1809 gastric patients. Our study showed that high LVD was associated with OS in a univariate analysis of seven studies. However, only two studies (Cosxkun U [20] and Park KH [21]) examined the prognostic value of LVD for DFS. Cosxkun U *et al.* [20] found that the median survival time of patients with high LVD was 31 months compared with 43 months for the low LVD group. A univariate analysis using the Kaplan-Meier survival method indicated that low LVD was significantly associated with better DFS (log-rank test, *P* = 0.04). Park KH [21] *et al.* compared 38 low-LVD patients with gastric cancer with 28 high-LVD ones and found that high LVD was correlated with advanced TNM stage and a poor prognosis according to the Kaplan-Meier method (log-rank test, *P* = 0.037). The results of both studies indicated that high levels of LVD could act as significant prognostic markers of poor DFS.

Previous studies have used meta-analysis to determine the characterization and impact of LVD markers in non-small cell lung carcinoma, head and neck squamous cell carcinoma, breast cancer, colorectal cancer, and melanoma [24–28]. The results collectively showed that high LVD was associated with an unfavorable prognosis. Lymphatic metastasis is common in the digestive system. Thus, we conducted a meta-analysis of LVD in gastric cancer. The prognosis for gastric cancer is generally poor because tumors often metastasize during the late stages of carcinoma. Apparently, the lymphatic vessel system plays a crucial role in the progress of gastric cancer. However, the lymphatic drainage of the stomach is intricate. Tumor lymphatics have been implicated in metastasis, and this system subsequently influences the survival rate [29]. The correlation between LVD and prognosis in gastric cancer has not yet been reported. However, peritumoral LVD in malignant tumors is suspected to actively reduce the survival time of cancer patients [30]. Our systematic review and meta-analysis investigated the correlation between peritumoral LVD and survival status in gastric cancer. In the present meta-analysis, 9 pooled studies were included [15–23]. The current finding showed that the high peritumoral LVD indicates a poor prognosis. Hence, the results of the sensitivity analyses indicated that the present study was stable, thereby confirming the reliability of this conclusion. These outcomes were based on prospective studies, which provided evidence for our analysis.

Lymph node metastasis is associated with distant metastasis and recurrence. Lymphangiogenesis contributes to tumor cell metastasis. Therefore, the spread of tumors through the lymphatic vessels plays a decisive role in the distant metastasis and recurrence of tumors. Recently, several studies have researched the molecular mechanism and biological characteristic of lymphangiogenesis, but the verdict remains unclear. VEGF-C and VEGF-D expression are positively associated with LVD [31]. Chen *et al.* proposed that VEGF-C and VEGF-D may participate in Akt/mTOR pathway and efficiently regulate lymphangiogenesis of gastric cancer [32]. Peng *et al.* suggested that SPARC could inhibit lymphangiogenesis in ovarian cancer by reducing VEGF-C and VEGF-D expression [33]. Park *et al.* indicated that PROX1 facilitated tumorigenesis and progression by inducing lymphangiogenesis in gastric cancer [34]. CXCL1 is secreted by lymphatic endothelial cells. Wang *et al.* [35] found that CXCL1 expression was elevated in gastric cancer and suggested that CXCL1 facilitates the migration of gastric cancer through integrin β1-FAK-AKT signaling. Although the mechanism of lymphangiogenesis and metastasis is not clear at present, LVD is still recognized as a significant predictor in patients with gastric cancer.

There are some limitations to this study. First, the number of included studies was small, as was the sample sizes of most of the studies. The study by Cao F *et al.* accounted for overwhelming majority of the samples [16]. With 1072 samples, the Cao F *et al.* study was larger than all the other studies combined (*n* = 737). Moreover, only two articles recorded the prognostic association between DFS and LVD. A quantitative analysis of only two studies is less effective than when more studies are included. Second, the definitions of LVD differed among the studies. The major differences include patient race, staining techniques, and antibody types. Other reasons include the use of different counting methods to evaluate LVD. Because D2-40 has been extensively used for clinicopathology, studies concerning VEGF-C, VEGF-D or VEGFR-3 were not included in this study. Thus, we selected peritumoral, but not intratumoral, LVD to analyze the pooled HRs. However, this analysis did not provide strong evidence concerning the impact of LVD on the prognosis of gastric cancer patients. Third, the HR data from the univariate analysis were extrapolated from survival curves, and the unsatisfactory inaccuracy of the survival rates could contribute to potential bias. Finally, the LVD cut-off value used in each study varied, and the high and low LVD levels were relative in each study, leading to heterogeneity in the present study. Moreover, some missing data was unavoidable, and only published data obtained from 5 authoritative databases were included.

Despite the limitations of this meta-analysis, we can conclude that high LVD indicated a poor prognosis for OS in gastric cancer patients and poor DFS in a small number of studies. To further investigate the clinical value of LVD, larger research studies and more reliable prognostic information are needed. When sufficient data on clinicopathological parameters with higher patient numbers and different LVD levels are available, further research concerning the influence of LVD in gastric cancer can be conducted.

## CONCLUSIONS

This comprehensive meta-analysis shows that high LVD is closely related to poor prognosis in gastric malignancy.

## MATERIALS AND METHODS

### Search strategy and study selection

The electronic databases EMBASE, PubMed, Cochrane Library, Chinese National Knowledge Infrastructure (CNKI) and Wanfang Data were searched (the latest search was updated in April 2017). The search strategy included the terms “gastrointestinal OR gastric OR stomach”, “cancer OR carcinoma OR tumor OR neoplasm OR malignan*”, and “lymphatic microvessel density OR LVD”. No language limitation was applied. The reference lists of the relevant publications were assessed.

### Inclusion criteria

The following inclusion criteria were generated to filter the studies included in this meta-analysis: (1) full research paper that directly evaluated LVD in gastric cancer patients; (2) results that included survival information (overall survival (OS) and/or recurrence-free survival (RFS); (3) immunohistochemistry (IHC) was used to evaluate LVD; (4) HR and 95% CI could be directly acquired from the paper or indirectly calculated through information from the article; and (5) the latest article was included when the same investigated cohort was published in different articles. Four independent authors (Wen-ting Huang, Chun-qin Huang, Hong Zhou, Fang-lin Wei) applied the inclusion criteria to assess the eligibility of the retrieved articles.

### Data extraction, critical appraisal and quality assessment

Four authors (Wen-ting Huang, Chun-qin Huang, Hong Zhou, Fang-lin Wei) independently extracted the data from all eligible studies, including the following items: first author's name, publication year, country, number of patients, test method and survival data. If a study contained the results of both univariate and multivariate analyses, both were included in our meta-analysis to obtain a more precise result. Controversial problems were resolved through discussion and consensus.

The quality assessment and the risk of bias of the included studies were determined using the NOS criteria [36]. The scale examines three factors - selection, comparability and outcome. The maximum score is nine, a score ≥6 indicates a high-quality study.

### Statistical analysis

Stata statistical software version 12.0 (Stata Corporation, College Station, TX) was used in the meta-analysis. The HR and 95% CI for the high- and low-LVD survival distributions were determined to estimate the LVD index and the survival data. Overall survival (OS) or disease-free survival (DFS) curves were estimated using the Kaplan-Meier (K-M) method. If HR and/or 95% CI were not directly reported in the studies, the K-M survival curves were read using Engauge Digitizer software version 4.1 (http://digitizer.sourceforge.net/) [37–39].

Statistical heterogeneity was estimated using Cochrane's *Q* test (chi-squared test; Chi^2^) and inconsistency (*I*^2^). Of the nine included studies, five used accurate values as a cut-off. The other studies used median, mean and complex score (Fromowitz methods) as cut-offs. Other publications have used a random-effects model to address the use of various cut-off values in the studies included in a meta-analysis [24, 40]; hence, we also used the random-effect model in our meta-analysis to reduce the heterogeneity. A sensitivity analysis was conducted to determine which studies contributed to the heterogeneity. By convention, HR > 1 stood for worse survival for the high-LVD patient group. Statistical significance was observed for the impact of LVD on survival when the 95% CI did not overlap 1. A significant two-way *P* value for comparison was defined as *P* ≤ 0.05.

Publication bias was assessed using Begg's funnel plot method [41] in this meta-analysis. If no publication bias was observed, *P >* 0.05 was obtained, and the graph showed a symmetrical inverted funnel. In contrast, if a publication bias was observed, *P* ≤ 0.05 was obtained, and a skewed and asymmetrical plot was shown.

## Abbreviations

LVD: lymphatic microvessel density
HR: hazard ratio
OS: overall survival
DFS: disease-free survival
RFS: recurrence-free survival
LYVE-1: lymphatic vessel endothelial receptor 1
CNKI: Chinese National Knowledge Infrastructure
IHC: immunohistochemistry
CI: confidence intervals
NOS: Newcastle-Ottawa Quality Assessment Scale
